# Exercise rehabilitation adherence and associated factors in patients aged ≥75 years with hemorrhagic stroke: a mixed-methods study

**DOI:** 10.3389/fneur.2026.1861509

**Published:** 2026-09-15

**Authors:** Yan Wang, Lan Lu, Wei Wei

**Affiliations:** The First Affiliated Hospital of Guangxi Medical University, Nanning, China

**Keywords:** aged, exercise therapy, hemorrhagic stroke, self efficacy, treatment adherence and compliance

## Abstract

**Background:**

Patients aged ≥75 years with hemorrhagic stroke are at high risk of functional impairment and poor recovery. Exercise rehabilitation is essential for restoring physical function, but adherence to exercise rehabilitation may be associated with multiple factors in this population. This study aimed to investigate exercise rehabilitation adherence and its associated factors among patients aged ≥75 years with hemorrhagic stroke using an explanatory sequential mixed-methods approach.

**Methods:**

A total of 254 patients aged ≥75 years with hemorrhagic stroke were recruited by convenience sampling between July 2024 and May 2025. Data were collected using a general information questionnaire, the Stroke Functional Exercise Adherence Scale, the Stroke Self-Efficacy Questionnaire, and the Mishel Uncertainty in Illness Scale. Univariate analysis and multinomial logistic regression were conducted to identify factors associated with adherence. Subsequently, 13 patients were purposively selected for semi-structured interviews to explore the experiences underlying the quantitative findings. Qualitative data were analyzed using Colaizzi’s seven-step method.

**Results:**

The mean adherence score was 37.97 ± 10.57. Overall, 20.87, 29.53, and 49.60% of participants demonstrated low, moderate, and high adherence, respectively. Multinomial logistic regression showed that higher rehabilitation self-efficacy was associated with lower odds of low adherence. In contrast, greater illness uncertainty and disease duration ≤6 months were associated with higher odds of low adherence. Greater illness uncertainty, male sex, and disease duration ≤6 months were also associated with moderate adherence. Qualitative analysis identified three themes underlying adherence: cognition and attitudes toward exercise rehabilitation, psychological and emotional disturbances, and external support.

**Conclusion:**

Exercise rehabilitation adherence varied among patients aged ≥75 years with hemorrhagic stroke and was associated with disease duration, rehabilitation self-efficacy, illness uncertainty, and sex. The qualitative findings further indicated that patients’ cognition and attitudes, psychological and emotional experiences, and external support shaped their engagement in rehabilitation. These findings highlight the potential value of tailored interventions addressing these multidimensional factors to support engagement in exercise rehabilitation among this vulnerable population.

## 1 Introduction

Hemorrhagic stroke is a major cause of death and disability worldwide, imposing a substantial burden on patients, families, and healthcare systems (1, 2). Intracerebral hemorrhage (ICH) and subarachnoid hemorrhage (SAH) are the two main subtypes of hemorrhagic stroke. In China, the burden of ICH remains substantial, with approximately 3.12 million incident cases and 913,023 deaths reported in 2021 (3). A systematic review and meta-analysis showed that the incidence of ICH increases markedly with age, rising from about 2 per 100,000 in individuals <45 years to 166 per 100,000 in those aged 75–84 years (4). According to commonly used age classifications, older adults can be categorized as young-old (65–74 years), old-old (75–84 years), and oldest-old (≥85 years) (5).

Hemorrhagic stroke frequently causes motor and functional impairments that affect mobility and activities of daily living (6). Exercise rehabilitation is a core component of post-stroke rehabilitation, playing an important role in improving motor function and activities of daily living (7). Post-stroke exercise rehabilitation typically includes upper- and lower-limb training, muscle strengthening, balance and gait training, and task-specific functional practice (8). Patients’ adherence is an important aspect of rehabilitation, defined as the extent to which they follow prescribed rehabilitation recommendations and engage in planned rehabilitation activities (9). Older adults are often characterized by reduced physiological reserve, frailty, and multimorbidity, which may limit their participation in rehabilitation and their ability to engage in regular rehabilitation activities (10–12). However, evidence regarding exercise rehabilitation adherence among very old adults with hemorrhagic stroke remains limited.

Previous studies of exercise rehabilitation adherence after stroke have largely used either quantitative or qualitative methods, with limited integration of quantitative findings and patients’ experiences. However, few studies have examined exercise rehabilitation adherence and its associated factors in patients aged 75 years and older with hemorrhagic stroke, particularly by combining quantitative findings with patients’ experiences. Given the marked increase in ICH incidence among individuals aged 75 years and older, this population warrants particular attention in stroke management and rehabilitation. Therefore, the following research questions were addressed: (1) What is the level of exercise rehabilitation adherence among patients aged ≥75 years with hemorrhagic stroke receiving care at a tertiary hospital in Guangxi, China? (2) Which factors are associated with exercise rehabilitation adherence, and how do patients’ experiences and perceptions help explain these associations? Accordingly, this study aimed to (1) assess exercise rehabilitation adherence among patients aged ≥75 years with hemorrhagic stroke; (2) to identify factors associated with adherence; and (3) to explore patients’ experiences and perceptions that may help explain the quantitative findings using an explanatory sequential mixed-methods approach.

## 2 Materials and methods

This study used an explanatory sequential mixed-methods design to investigate exercise rehabilitation adherence and associated factors among patients aged ≥75 years with hemorrhagic stroke. A cross-sectional survey was followed by semi-structured interviews with purposively selected participants from the quantitative sample after discharge. Quantitative findings guided participant selection and interview guide development, and qualitative findings were integrated during interpretation to explain the quantitative associations. The quantitative phase of the study was reported in accordance with the STROBE guidelines.

### 2.1 Participants

Quantitative phase: Participants were consecutively recruited from hospitalized patients receiving medical and nursing care in the Department of Neurology at the First Affiliated Hospital of Guangxi Medical University, Nanning, China, between July 2024 and May 2025. The inclusion criteria were: (1) hemorrhagic stroke confirmed by neuroimaging and consistent with the relevant diagnostic criteria (13); (2) age ≥75 years; (3) ability to communicate effectively and understand the questionnaire instructions; and (4) provision of informed consent. Before enrollment, trained researchers assessed communication and questionnaire comprehension through brief verbal communication and by explaining the questionnaire purpose, instructions, and response options. Participants were considered eligible if they could understand the instructions and provide coherent and appropriate responses to the questionnaire items. Exclusion criteria were: (1) severe comorbid conditions, such as malignant tumors or severe hepatic or renal failure; and (2) unstable clinical conditions that prevented participation in the survey. Participants with patterned or invalid responses, substantial missing data, withdrawal, or severe complications or death during the study period were excluded from the final analysis.

Qualitative phase: Following completion of the quantitative phase, purposive sampling with maximum variation was used to select eligible participants from the quantitative sample for semi-structured interviews conducted between July and August 2025. Eligible participants were identified from the quantitative dataset according to predefined variation criteria and invited to participate in the interviews. Maximum variation was sought across exercise rehabilitation adherence level, duration since stroke onset, age, sex, and education level, and other relevant clinical characteristics were also considered. Data saturation was defined as the point at which consecutive interviews yielded no new codes, categories, or meaningful insights relevant to the research questions. The interviewer and two researchers jointly reviewed emerging data and discussed whether additional interviews were needed. Recruitment was discontinued when consensus was reached that saturation had been achieved.

### 2.2 Instruments

A self-designed questionnaire was used to collect demographic and clinical information, including sex, age, education level, living arrangement, medical insurance type, residence, and disease duration.

The Stroke Functional Exercise Adherence Scale developed by Lin et al. (14) was used. The scale consists of 14 items across three dimensions: adherence related to physical participation in exercise, adherence related to seeking timely advice during exercise, and adherence related to monitoring exercise effects. Items are rated on a 4-point Likert scale, with a total score ranging from 14 to 56, where higher scores indicate better adherence. The adherence rate is calculated as actual score / maximum possible score × 100% and categorized as high (>75%), moderate (50–75%), or low (<50%) adherence. The scale demonstrated good reliability with a Cronbach’s α of 0.901.

Rehabilitation self-efficacy was assessed using the Chinese version of the Stroke Self-Efficacy Questionnaire translated and validated by Li et al. (15). The scale consists of 11 items across two dimensions: activities of daily living and self-management. Scores range from 0 to 110, with higher scores indicating greater confidence in performing rehabilitation-related behaviors. The scale demonstrated excellent internal consistency, with a Cronbach’s α coefficient of 0.969.

The Chinese version of the Mishel Uncertainty in Illness Scale (MUIS), translated by Sheu (16). The scale contains 33 items across four dimensions: ambiguity, complexity, lack of information, and unpredictability. Item 15 is not scored. Items are rated on a 5-point Likert scale, with total scores ranging from 32 to 160; higher scores indicate greater illness uncertainty. The scale demonstrated good reliability (Cronbach’s *α* = 0.91). Neither the Stroke Self-Efficacy Questionnaire nor the Chinese version of the Mishel Uncertainty in Illness Scale provides established cutoff values for categorizing scores into different levels. Therefore, both measures were analyzed as continuous variables, with higher scores indicating greater rehabilitation self-efficacy and illness uncertainty, respectively.

### 2.3 Interview guide for the qualitative phase

The semi-structured interview guide was developed based on key findings from the quantitative phase, relevant literature on exercise rehabilitation adherence and its barriers and facilitators among older adults with stroke, and research team discussions. The guide explored participants’ experiences and perceptions of exercise rehabilitation, reasons for adherence or non-adherence, barriers and facilitators, and perceived rehabilitation support needs. The guide was refined through team discussions before the interviews and is provided as Supplementary materials.

### 2.4 Data collection and quality control

Investigators received standardized training before data collection. After the study purpose and procedures were explained and written informed consent was obtained, quantitative data were collected through face-to-face question-and-answer interviews, with questionnaires completed by the researchers.

Selected participants were contacted after discharge through outpatient follow-up visits, telephone calls, or WeChat. Before each interview, the study purpose and procedures were explained, and willingness to participate and permission for audio recording were reconfirmed. Each interview was conducted using a semi-structured, open-ended interview guide and lasted approximately 30–40 min. Recordings were transcribed and cross-checked independently by two researchers within 24 h, and unclear information was verified with participants.

### 2.5 Data analysis

#### 2.5.1 Quantitative data analysis

Data were independently checked and organized by two researchers using Microsoft Excel 2016 and analyzed using SPSS version 26.0. Continuous variables were assessed for distributional characteristics and are presented as mean ± standard deviation (SD) for normally distributed data. Continuous variables included age, rehabilitation self-efficacy, and illness uncertainty scores, whereas categorical variables included demographic and clinical characteristics such as sex, education level, living arrangement, and disease duration. Differences in continuous variables among the low-, moderate-, and high-adherence groups were examined using one-way analysis of variance (ANOVA). Categorical variables are presented as frequencies and percentages and were compared among the three adherence groups using the chi-square (χ^2^) test.

Univariate analyses were first conducted to examine the associations between potential factors associated with exercise rehabilitation adherence. Variables with *p* < 0.05 in the univariate analyses were subsequently entered into the multinomial logistic regression model. Disease duration was categorized as ≤6 months and >6 months based on clinical considerations, reflecting clinically meaningful differences between earlier and later stages of stroke recovery. The high-adherence group was specified as the reference category for the dependent variable, and reference categories for categorical predictors were specified *a priori*. Multicollinearity among predictors was assessed using tolerance and variance inflation factor (VIF). Tolerance values >0.10 and VIF values <10 were considered indicative of the absence of serious multicollinearity. The final model was evaluated based on model convergence, the likelihood-ratio chi-square test, and pseudo-R^2^ statistics. The Wald test was used to assess the statistical significance of individual regression coefficients, and odds ratios (ORs) with 95% confidence intervals (CIs) were reported. All statistical tests were two-sided, and *p* < 0.05 was considered statistically significant.

#### 2.5.2 Qualitative data analysis

NVivo 11.0 was used to organize and manage the qualitative data. Interview transcripts were analyzed using Colaizzi’s seven-step phenomenological method (17). The researchers (1) repeatedly read the transcripts to gain an overall understanding; (2) identified and extracted significant statements related to exercise rehabilitation adherence; (3) formulated meanings from these statements; (4) grouped related meanings into theme clusters; (5) integrated the clusters into an exhaustive description; (6) condensed the description into the fundamental structure of the phenomenon; and (7) reviewed the final thematic structure against the original transcripts to ensure that it accurately reflected participants’ experiences. Two researchers independently coded the transcripts and compared their interpretations. Any discrepancies in coding or theme development were discussed between the two researchers and further reviewed with a third researcher until consensus was reached.

### 2.6 Ethical considerations

The study was conducted in accordance with the Declaration of Helsinki and was approved by the Ethics Committee of the First Affiliated Hospital of Guangxi Medical University (2024-S025-01). All participants provided written informed consent before enrollment in the quantitative phase and were informed of the voluntary nature of participation, confidentiality of their information, and their right to withdraw at any time without affecting their medical care. Before the qualitative interviews, participants’ consent was reconfirmed. All data were anonymized and used solely for research purposes.

## 3 Results

### 3.1 Quantitative results

#### 3.1.1 Participants’ baseline characteristics

A total of 254 patients aged ≥75 years with hemorrhagic stroke were included (mean age 82.99 ± 3.32 years), including 125 males (49.21%) and 129 females (50.79%). 124 (48.82%) had medical insurance and 130 (51.18%) were self-paying. 133 (52.36%) had junior high school education or below and 121 (47.64%) had high school education or above. 126 (49.61%) had a disease duration ≤6 months and 128 (50.39%) > 6 months. 141 (55.51%) lived in urban areas and 113 (44.49%) in rural areas. 134 (52.76%) lived alone. Stroke episodes were first (32.07%), second (33.86%), and ≥3 (33.07%).

#### 3.1.2 Levels of exercise rehabilitation adherence

The mean adherence score was 37.97 ± 10.57, with an overall adherence rate of 67.80%. 20.87% of patients showed low adherence, 29.53% moderate adherence, and 49.60% high adherence. Scores for each dimension and other related scales are presented in Table 1.

**Table 1:** Scale scores of patients aged ≥75 years with hemorrhagic stroke (*n* = 254).

Item	Score (mean±SD)	Item mean (mean±SD)	Adherence [*n* (%)]		
			Low	Moderate	High
Stroke functional exercise adherence					
Physical exercise	23.57 ± 6.06	2.95 ± 0.76	24 (9.45)	87 (34.25)	143 (56.30)
Exercise monitoring	7.53 ± 2.77	2.51 ± 0.92	80 (31.50)	69 (27.16)	105 (41.34)
Seeking advice	6.85 ± 2.61	2.28 ± 0.87	100 (39.37)	82 (32.28)	72 (28.35)
Total score	37.95 ± 10.54	2.72 ± 0.76	53 (20.87)	75 (29.53)	126 (49.60)
Stroke self-Efficacy	54.53 ± 10.53	4.96 ± 0.96			
Illness uncertainty	65.22 ± 10.59	2.17 ± 0.35			

SD, standard deviation; n, number of participants; %, percentage.

#### 3.1.3 Univariate analysis of participant characteristics

No significant differences were found among adherence groups in age, education level, or household income (*p* > 0.05). Significant differences were observed in sex, disease duration, rehabilitation self-efficacy, and illness uncertainty (*p* < 0.05) (Table 2).

**Table 2:** Univariate analysis of exercise rehabilitation adherence among patients with hemorrhagic stroke (*n* = 254).

Variables	Low (*n* = 53)	Moderate (*n* = 75)	High (*n* = 126)	χ^2^*/F*	*p*
Sex				10.690	0.005
Male	31 (58.49%)	45 (60.00%)	49 (38.89%)		
Female	22 (41.51%)	30 (40.00%)	77 (61.11%)		
Age (years)	82.69 ± 3.38	83.14 ± 3.74	82.81 ± 3.02	0.342	0.711
Payment method				3.192	0.203
Insurance	23 (43.40%)	43 (57.33%)	58 (46.03%)		
Self-paid	30 (56.60%)	32 (42.67%)	68 (53.97%)		
Education level				0.009	0.996
Junior high or below	28 (52.83%)	39 (52.00%)	66 (52.38%)		
High school or above	25 (47.17%)	36 (48.00%)	60 (47.62%)		
Disease duration				18.225	<0.001
≤6 months	36 (67.92%)	44 (58.67%)	46 (36.50%)		
>6 months	17 (32.08%)	31 (41.33%)	80 (63.50%)		
Residence				2.560	0.278
Urban	30 (56.60%)	36 (48.00%)	75 (59.52%)		
Rural	23 (43.40%)	39 (52.00%)	51 (40.48%)		
Monthly household income (RMB)				3.657	0.723
<2000 yuan	15 (28.30%)	22 (29.33%)	37 (29.37%)		
2000 ~ <4,000 yuan	12 (22.64%)	20 (26.67%)	31 (24.60%)		
4,000 ~ <10,000 yuan	17 (32.08%)	18 (24.00%)	26 (20.63%)		
>10,000 yuan	9 (16.98%)	15 (20.00%)	32 (25.40%)		
Living arrangement				2.246	0.325
Living alone	24 (45.28%)	44 (58.67%)	66 (52.38%)		
Not living alone	29 (54.72%)	31 (41.33%)	60 (47.62%)		
Number of stroke episodes				1.979	0.740
First episode	16 (30.19%)	23 (30.67%)	45 (35.71%)		
Two episode	23 (43.40%)	23 (30.67%)	40 (31.75%)		
≥3 episodes	14 (26.41%)	29 (38.66%)	41 (32.54%)		
Illness uncertainty	68.37 ± 9.71	67.04 ± 11.38	62.82 ± 9.95	6.996	<0.001
Rehabilitation self-efficacy	49.56 ± 12.42	54.49 ± 9.90	56.65 ± 9.93	7.089	0.001

*n*, number of participants; %, percentage; χ^2^, chi-square statistic; F, F statistic; P, p value; RMB, Renminbi (Chinese currency).

#### 3.1.4 Factors associated with different levels of exercise rehabilitation adherence

Variables identified as significant in the univariate analyses were entered into the multinomial logistic regression model as predictors. The coding of variables included in the model is presented in Table 3. The likelihood-ratio test indicated that the model provided a significantly better fit than the intercept-only model (likelihood-ratio χ^2^ = 51.380, df = 8, *p* < 0.001). The pseudo-R^2^ values were 0.183 (Cox and Snell), 0.210 (Nagelkerke), and 0.098 (McFadden), suggesting a satisfactory model fit.

**Table 3:** Coding of variables included in the multinomial logistic regression model.

Variable	Coding
Sex	Male = 0, Female = 1
Disease duration	≤6 months = 0, >6 months = 1
Illness uncertainty	Continuous
Rehabilitation self-efficacy	Continuous

Using the high-adherence group as the reference category, higher rehabilitation self-efficacy was associated with lower odds of low adherence (OR = 0.936, 95% CI: 0.903–0.970, *p* < 0.001), whereas greater illness uncertainty was associated with higher odds of low adherence (OR = 1.046, 95% CI: 1.011–1.083, *p* = 0.009). Participants with a disease duration of ≤6 months had higher odds of low adherence (OR = 3.463, 95% CI: 1.687–7.109, *p* = 0.001) than those with a disease duration of >6 months. Sex was not significantly associated with low adherence (*p* = 0.136).

Compared with the high-adherence group, greater illness uncertainty was associated with higher odds of moderate adherence (OR = 1.035, 95% CI: 1.005–1.066, *p* = 0.022). Male participants had higher odds of moderate adherence than female participants (OR = 2.020, 95% CI: 1.102–3.702, *p* = 0.023), and participants with a disease duration of ≤6 months had higher odds of moderate adherence than those with a disease duration of >6 months (OR = 2.320, 95% CI: 1.269–4.242, *p* = 0.006). Rehabilitation self-efficacy was not significantly associated with moderate adherence (*p* = 0.249). These results are shown in Table 4.

**Table 4:** Multinomial logistic regression analysis of exercise rehabilitation adherence among patients with hemorrhagic stroke.

Item	*β*	SE	Wald	*p*	OR	95% CI
Low vs. High						
Sex (with female as the reference)						
Male	0.537	0.361	2.217	0.136	1.711	0.844 ~ 3.470
Disease duration (with > 6 months as the reference)						
≤6 months	1.242	0.367	11.463	0.001	3.463	1.687 ~ 7.109
Self-efficacy	−0.066	0.018	13.133	<0.001	0.936	0.903 ~ 0.970
Illness uncertainty	0.045	0.017	6.769	0.009	1.046	1.011 ~ 1.083
Moderate vs. High						
Sex (with female as the reference)						
Male	0.703	0.309	5.175	0.023	2.020	1.102 ~ 3.702
Disease duration (with > 6 months as the reference)						
≤6 months	0.842	0.308	7.469	0.006	2.320	1.269 ~ 4.242
Self-efficacy	−0.017	0.015	1.330	0.249	0.983	0.954 ~ 1.012
Illness uncertainty	0.035	0.015	5.232	0.022	1.035	1.005 ~ 1.066

*β*, regression coefficient; SE, standard error; Wald, Wald statistic; *p*, *p* value; OR, odds ratio; CI, confidence interval. The high adherence group was used as the reference category in the multinomial logistic regression model.

### 3.2 Qualitative results

The 13 participants included 7 men and 6 women, aged 76 to 87 years. The duration since stroke ranged from 2 to 12 months. Participants represented low, moderate, and high levels of exercise rehabilitation adherence and varied in educational and functional characteristics. Their characteristics are shown in Table 5.

**Table 5:** Characteristics of interview participants.

Participant ID	Sex	Age (years)	Education	Disease duration (months)	First-ever stroke	Living arrangement	Adherence level
P1	Male	77	Junior high	3	Yes	Living alone	High
P2	Female	78	Primary school	8	No	Living alone	Moderate
P3	Male	80	High school	2	Yes	Living with children	High
P4	Female	79	Illiterate	10	No	Living with children	Low
P5	Male	79	University	4	Yes	Living alone	Moderate
P6	Female	81	Junior high	7	No	Living with spouse	Low
P7	Male	84	High school	6	Yes	Living with spouse	High
P8	Female	87	Primary school	12	No	Living with children	Moderate
P9	Male	80	Junior high	5	Yes	Living with family	High
P10	Female	79	Illiterate	9	No	Living alone	Low
P11	Male	76	High school	3	Yes	Living with spouse	High
P12	Female	82	Primary school	11	No	Living with children	Moderate
P13	Male	83	University	2	Yes	Living with family	High

ID, participant identification.

Analysis of the interview data generated three themes regarding exercise rehabilitation adherence among older adults with hemorrhagic stroke. These themes are: (1) Perceptions and attitudes toward exercise rehabilitation; (2) Psychological and emotional experiences in stroke recovery; and (3) External support and rehabilitation experiences.

#### 3.2.1 Theme 1: Perceptions and attitudes toward exercise rehabilitation

##### 3.2.1.1 Limited understanding of exercise rehabilitation

Several participants described a limited understanding of exercise rehabilitation, particularly during the early stages of recovery. Rehabilitation was sometimes perceived as a supplementary activity For example, P1 described rehabilitation exercise as “similar to square dancing,” while P3 believed that recovery mainly depended on medication during hospitalization.

Participants also reported difficulties in understanding or remembering rehabilitation instructions. P8 stated that they could not remember “how or how much to exercise,” whereas P2 indicated that the principles of rehabilitation were not sufficiently clear. In some cases, uncertainty about how to perform exercises was accompanied by concerns about exercising incorrectly. P4 described avoiding exercise because of uncertainty about whether the exercises were being performed correctly. Similarly, P3 and P12 described performing exercises incorrectly and reported difficulties in understanding instructions or receiving ongoing guidance and feedback.

##### 3.2.1.2 Limited expectations and confidence regarding recovery

Participants frequently described uncertainty about their recovery and limited confidence in the potential benefits of rehabilitation. Some participants perceived advanced age as a limitation to functional recovery and expressed low expectations regarding the benefits of exercise. P10 explained, “I practiced for more than 10 days but saw no change… maybe I’m too old to recover.”

Others reported that their initial expectations decreased when improvements were slower than anticipated. P12 stated, “I think this is just how it will be; if exercise worked, it would have helped already.” Some participants also appeared to expect relatively rapid changes following rehabilitation. When noticeable improvements did not occur within a short period, they described reducing or discontinuing exercise (P4, P6). Family members’ views were also mentioned in some accounts. For example, P8 reported that family members believed that exercise would make little difference and described being reluctant to continue rehabilitation.

##### 3.2.1.3 Passive engagement and inconsistent adherence

Participants described rehabilitation participation as passive or inconsistent in several ways. Some participants reported engaging in exercises mainly when reminded by healthcare professionals or family members (P6, P8, P9). Adherence was also interrupted by everyday distractions or reduced motivation. Although some participants recognized the value of rehabilitation, they reported stopping or selectively performing exercises during the rehabilitation process (P2, P7, P11).

#### 3.2.2 Theme 2: Psychological and emotional experiences in stroke recovery

##### 3.2.2.1 Negative emotional experiences

Participants described a range of negative emotional experiences in the course of stroke recovery, including anxiety, depressive feelings, anger, helplessness, and emotional withdrawal. These experiences were often discussed in relation to sudden functional changes, slow recovery, or uncertainty about future outcomes.

Some participants described feeling discouraged when recovery was slower than expected or when comparing their progress with that of others. P4, P11, and P13 expressed a sense of hopelessness or reduced motivation in these circumstances. Other participants described withdrawal from communication and reduced initiative in rehabilitation activities (P5, P10). Some participants also described emotional indifference or behavioral withdrawal in the context of prolonged functional limitations and reduced mobility (P6).

##### 3.2.2.2 Concerns about rehabilitation outcomes and safety

Uncertainty about rehabilitation outcomes and concerns about possible risks during exercise were also frequently described. Some participants worried that excessive activity or incorrect movements might cause harm or worsen their condition. P4, P8, and P12 expressed concerns or doubts about the safety and potential benefits of exercise rehabilitation.

In some cases, participants reported performing exercises symbolically or minimally without fully engaging in the recommended rehabilitation plan (P2, P7). P3 also described experiencing discomfort or perceived worsening of symptoms during rehabilitation and subsequently becoming more reluctant to continue exercising.

#### 3.2.3 Theme 3: External support and rehabilitation experiences

##### 3.2.3.1 Family encouragement and companionship

Family members were frequently mentioned in participants’ accounts of rehabilitation. Encouragement, companionship, assistance with exercises, and ongoing attention were described as sources of support during training. Several participants reported that family members’ involvement encouraged them to continue exercising and provided practical assistance when needed (P3, P6, P9, P13). Conversely, some participants described limited family support or a lack of assistance. P1 and P5 reported feeling neglected or lacking someone to accompany or assist them during rehabilitation.

##### 3.2.3.2 Professional guidance and encouragement

Participants also described the influence of interactions with healthcare professionals on their rehabilitation experiences. Clear demonstrations, explanations of the purpose of exercises, and regular encouragement were reported as helpful in understanding how to perform rehabilitation exercises and in maintaining confidence during training (P1, P2, P4, P5, P7). In contrast, some participants described uncertainty about whether exercises were being performed correctly when instructions were insufficient or unclear. P8 described insufficient guidance, uncertainty about how to perform the exercises, and a tendency to avoid exercise.

##### 3.2.3.3 Access to appropriate rehabilitation resources

The participants also described the availability and accessibility of rehabilitation resources after discharge. Some participants reported that access to community rehabilitation services, home visits, or simple rehabilitation equipment supported their continued rehabilitation after discharge (P3, P9). Others mentioned that they had to travel long distances or had difficulty accessing rehabilitation services in their local communities (P2, P5). The participants also mentioned situations where the equipment was not suitable or the usage instructions were insufficient (P7).

### 3.3 Integration of quantitative and qualitative findings

Integration of the quantitative and qualitative findings provided a more comprehensive understanding of exercise rehabilitation adherence among older adults aged ≥75 years with hemorrhagic stroke. The quantitative analysis identified rehabilitation self-efficacy, illness uncertainty, disease duration, and sex as factors associated with different levels of adherence, while the qualitative findings provided contextual insights into participants’ experiences of rehabilitation. The integrated findings are illustrated in the conceptual framework (Figure 1).

**Figure 1:**
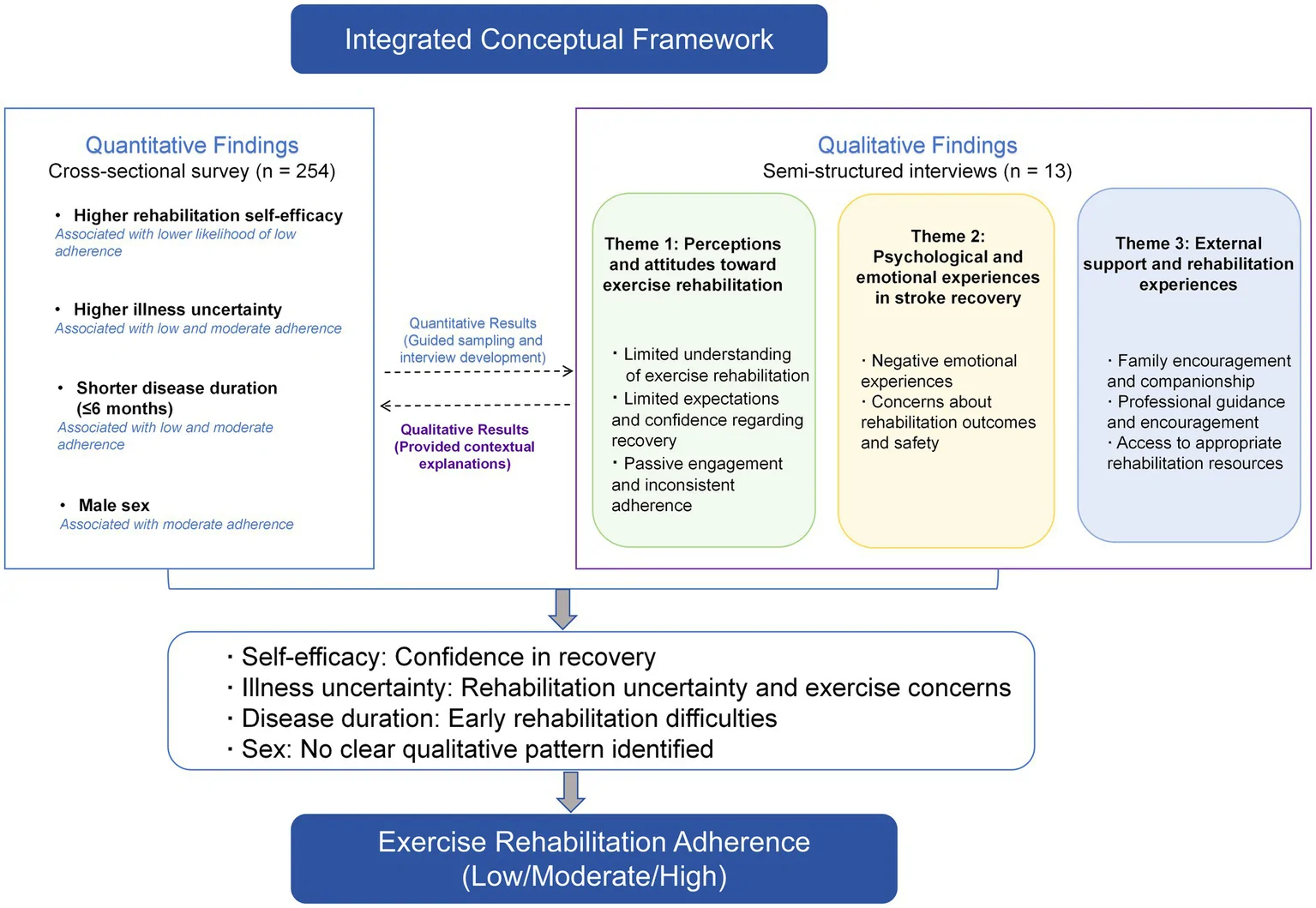
Integrated conceptual framework of factors associated with exercise rehabilitation adherence among older adults with hemorrhagic stroke. Note: *n*, number of participants. Quantitative associations were derived from multinomial logistic regression with the high-adherence group as the reference category.

Lower rehabilitation self-efficacy and higher illness uncertainty were associated with a greater likelihood of low adherence compared with high adherence. The qualitative findings complemented these associations by illustrating participants’ limited confidence in recovery, discouragement when improvement was slow or not immediately observable, uncertainty about rehabilitation outcomes, and concerns about exercise-related harm. These experiences were reflected in reduced motivation, avoidance, minimal engagement, or reluctance to persist with rehabilitation.

Participants with a disease duration of ≤6 months had higher odds of low adherence than those with a disease duration of >6 months. In the interviews, participants described difficulties in understanding rehabilitation instructions and remembering exercise procedures. Some were also unsure about how and how much to exercise. These findings provided additional information about the rehabilitation difficulties reported by participants. However, the qualitative data did not directly examine differences by disease duration. Male sex was associated with moderate adherence compared with high adherence in the quantitative analysis. The qualitative interviews did not identify a clear sex-related pattern that could explain this finding.

The qualitative findings provided further insight into several factors identified in the quantitative analysis. Participants’ accounts highlighted the role of recovery confidence, understanding of rehabilitation, and concerns about exercise participation. Their experiences also revealed challenges in learning and carrying out rehabilitation exercises after discharge. However, the interviews did not identify specific experiences that could account for the observed association between sex and adherence levels.

## 4 Discussion

### 4.1 Exercise rehabilitation adherence among patients aged ≥75 years with hemorrhagic stroke

The moderate level of exercise rehabilitation adherence in this study was consistent with findings from previous studies. Xing et al. (18) reported a mean adherence score of 38.41 ± 11.13 and an adherence rate of 68.6% among patients with acute stroke. Similarly, Lin et al. (19) found that 62.0% of community-dwelling stroke survivors adhered to physical activity and exercise (PAE). Gunnes et al. (20) further reported that, during an 18-month follow-up, 51.2–73.1% of patients with mild-to-moderate stroke maintained recommended daily physical activity levels, and 63.5–79.7% completed weekly structured exercise.

However, adherence appears to be substantially lower in rural settings. One previous study reported that only 17.8% of participants regularly engaged in PAE (21), a considerably lower rate than those reported in urban settings or contexts with greater access to healthcare and rehabilitation services. This difference may be related to contextual factors identified in previous research, such as limited rehabilitation resources, restricted access to rehabilitation services, insufficient health knowledge, and inadequate social support (19, 21, 22). These factors were not directly assessed in the present study. They may help explain the difference, but this interpretation should be treated with caution. Nevertheless, the finding that nearly half of participants had low or moderate adherence indicates that sustaining exercise rehabilitation remains challenging among very old adults with hemorrhagic stroke. Targeted support is needed to address modifiable barriers and facilitate the transition from hospital-based rehabilitation to long-term self-management.

### 4.2 Disease duration and illness uncertainty as barriers to exercise rehabilitation adherence

Patients with a disease duration of ≤6 months were more likely to demonstrate low adherence than those with a longer disease duration. This finding could reflect the challenges of maintaining engagement in rehabilitation during the early post-stroke period. Lin et al. (19) similarly reported that longer post-stroke duration was positively associated with physical activity adherence, suggesting that rehabilitation behaviors may become more established over time. However, findings from previous studies have been inconsistent. Tan et al. (23) reported a negative association between time since stroke and rehabilitation motivation, whereas Kobylańska et al. (24) found that delayed initiation of rehabilitation (>16 weeks) was associated with poorer functional improvement and participation. These conflicting findings indicate that the relationship between time since stroke and rehabilitation adherence is complex. Previous studies have found that adherence among AHS patients follows an “S-shaped” trajectory; it increases during hospitalization when professional support is available, declines after discharge, and potentially stabilizes in the absence of additional support (25). Wen et al. (26) further showed that adherence can change over time and may be improved through multimodal feedback and personalized digital interventions. Therefore, continued guidance and support throughout different stages of rehabilitation may be important for sustaining adherence.

Illness uncertainty was also independently associated with lower rehabilitation adherence. Qualitative findings provide further insight into this association, as participants often expressed concerns about recovery and uncertainty regarding the safety and benefits of exercise. Such uncertainty could contribute to anxiety and reduced engagement in rehabilitation activities (27, 28). Previous studies have shown that greater knowledge and understanding of stroke and its rehabilitation process are associated with better exercise adherence, particularly during the early post-stroke period (12). Moreover, illness perceptions can influence rehabilitation behaviors, functional outcomes, and psychological well-being (29, 30). Xing et al. (18) reported that mental health status was associated with rehabilitation adherence among patients with acute stroke, highlighting the relevance of psychological factors in rehabilitation planning. Groeneveld et al. (31) further found that negative changes in illness perceptions during the 3–12 months after stroke were associated with poorer physical and mental functioning and increased depressive symptoms. Collectively, these findings suggest that illness uncertainty represents a psychological barrier to rehabilitation engagement, particularly when patients lack clear expectations about recovery. Providing individualized information on recovery trajectories, clarifying exercise safety and purpose, and offering feedback on progress could help reduce uncertainty and support sustained rehabilitation participation.

### 4.3 Self-efficacy and the differential patterns of adherence

In this study, higher rehabilitation self-efficacy was associated with lower odds of low adherence; however, it did not significantly distinguish patients with moderate adherence from those with high adherence. One possible explanation is that self-efficacy is be related to patients’ motivation and confidence in engaging in rehabilitation activities. Previous studies have shown that post-stroke self-efficacy is associated with rehabilitation motivation and active participation, (19, 32–34). Lower self-efficacy can reduce confidence in performing rehabilitation exercises and may contribute to lower adherence (35). Among patients with moderate adherence, self-efficacy alone may not fully explain differences in adherence levels. Other factors, including physical function, family support, healthcare guidance, and rehabilitation resources, could also contribute to achieving higher adherence. Therefore, self-efficacy appears to be particularly relevant for identifying patients at risk of low adherence, whereas higher adherence likely requires broader clinical and environmental support.

The qualitative findings also supported this interpretation. Participants with higher adherence commonly described positive beliefs regarding the benefits of exercise and identified encouragement from family members and healthcare professionals as important facilitators of continued participation. These findings suggest that self-efficacy represents a psychological factor associated with rehabilitation engagement, while higher levels of adherence also involves functional and social factors.

### 4.4 The role of demographic and socioeconomic characteristics in adherence patterns

Demographic and socioeconomic characteristics showed different patterns across adherence levels. Male participants had higher odds of moderate adherence than high adherence, whereas sex was not significantly associated with low adherence. The qualitative findings did not provide a clear explanation for this sex-specific association. Further studies are needed to examine whether the association is related to differences in rehabilitation needs, support, or health behaviors. Previous studies have reported sex-related differences in health behaviors and treatment engagement, although these patterns vary across rehabilitation contexts (36, 37).

Although all participants were aged ≥75 years, age was not significantly associated with adherence. The restricted age range may have limited the ability to detect age-related differences. Moreover, among very old adults, functional ability, frailty, cognitive status, and comorbidity burden are likely to be more closely related to rehabilitation participation than chronological age in this age group. Education level and household income were also not significantly associated with adherence. Although lower education and socioeconomic status have been associated with poorer functional outcomes after stroke (38), their relationship with rehabilitation adherence may be less direct. The associations may be less apparent when family support, professional guidance, and clinical status play more immediate roles in rehabilitation participation. Overall, demographic and socioeconomic characteristics should therefore be interpreted alongside clinical and psychosocial factors when evaluating adherence among very old stroke survivors.

### 4.5 Integration of quantitative and qualitative findings

The integration of quantitative and qualitative findings provided a more comprehensive understanding of exercise rehabilitation adherence among very old adults with hemorrhagic stroke. The quantitative analysis identified several factors associated with adherence levels, including disease duration, illness uncertainty, self-efficacy, and sex. The qualitative findings provided further insights into these associations from patients’ perspectives.

The association between shorter disease duration and lower adherence was consistent with participants’ descriptions of uncertainty during the early recovery period. Participants often expressed concerns about their recovery progress and uncertainty about the safety and benefits of exercise. These experiences may help explain why greater illness uncertainty was associated with lower odds of achieving high adherence in the quantitative analysis. Similarly, the association between lower self-efficacy and low adherence was consistent with participants’ reports of limited confidence and concerns about rehabilitation. This pattern highlights the possible relevance of psychological factors among patients who struggle to engage in rehabilitation activities. A recent explanatory sequential mixed-methods study also identified disease duration and self-care ability as factors associated with home-based exercise rehabilitation adherence among stroke patients (39). However, the direction of the association with disease duration differed from our findings, which may reflect differences in age and stroke characteristics between the two studies. In our study, all participants were aged ≥75 years and had hemorrhagic stroke, whereas the previous study included a broader stroke population. These differences may also help explain why age was not associated with adherence in our study.

The qualitative findings also extended the quantitative results by highlighting the importance of family and professional support. Although these factors were not directly identified as significant correlates in the quantitative analysis, this finding is consistent with a mixed-methods study of patients with chronic heart failure (40). That study found that social support was associated with home-based exercise adherence and that self-efficacy played a chain-mediating role in this relationship. These findings suggest that adherence may reflect individual psychological factors and may also be associated with the social and rehabilitation environment. However, our qualitative findings did not directly explain the sex-specific association observed in the quantitative analysis. This finding requires further investigation. Overall, the mixed-methods findings suggest that psychological, clinical, and social factors may jointly shape exercise rehabilitation adherence among very old adults with hemorrhagic stroke.

### 4.6 Limitations

Several limitations should be considered when interpreting the findings of this study. First, the quantitative component used a cross-sectional design; therefore, the associations identified between sex, disease duration, rehabilitation self-efficacy, illness uncertainty, and exercise rehabilitation adherence cannot be interpreted as causal relationships. Longitudinal studies are needed to further examine the temporal relationships among these variables.

Second, the study was conducted in a single tertiary hospital in one region of China, and the sample size was relatively limited. In addition, all participants were aged ≥75 years, which may restrict the applicability of the findings to younger stroke populations or other healthcare settings. Future multicenter studies involving larger and more diverse samples are warranted to improve the generalizability of the findings.

Third, several key variables, including exercise rehabilitation adherence, rehabilitation self-efficacy, and illness uncertainty, were assessed using self-report questionnaires. These measures may be affected by recall bias and social desirability bias. Although standardized instructions were provided and researchers avoided influencing participants’ responses, assistance during questionnaire completion may still have introduced potential interviewer-related bias. Future studies could incorporate objective measures of rehabilitation participation to complement self-reported assessments. In addition, the Stroke Self-Efficacy Questionnaire and the Chinese version of the Mishel Uncertainty in Illness Scale do not provide established cutoff values for categorizing scores into different levels, which may limit the interpretability of the absolute scores. Future studies could incorporate objective measures of rehabilitation participation and establish clinically meaningful reference values to support the interpretation of these measures.

Fourth, limitations related to the qualitative component should also be acknowledged. Although the coding process involved independent coding by two researchers and discussion with a third researcher to reach consensus, some interpretive subjectivity may have remained during theme development. In addition, member checking with participants was not conducted, which may have limited further validation of the qualitative findings. Future studies should consider incorporating member checking to further enhance the credibility of qualitative research.

## 5 Conclusion

This mixed-methods study found that older adults aged ≥75 years with hemorrhagic stroke had moderate exercise rehabilitation adherence. Quantitative findings showed that adherence was associated with sex, disease duration, rehabilitation self-efficacy, and illness uncertainty, while qualitative findings indicated that adherence was shaped by the interplay of physiological status, psychological perceptions, and external support. These findings suggest that rehabilitation care should extend beyond physical and functional recovery to address patients’ psychological and social needs. Clinically, individualized interventions should focus on strengthening rehabilitation self-efficacy, reducing illness uncertainty, encouraging family involvement, and enhancing continuous professional support throughout the rehabilitation process. Future multicenter studies with larger and more diverse samples and longitudinal designs are warranted to further examine changes in rehabilitation adherence over time and their long-term effects on recovery outcomes.

## Data Availability

The datasets generated and/or analyzed in this study are not publicly available due to privacy and confidentiality considerations. They are available from the first and/or corresponding author upon reasonable request.
