# Supplementary material for: Exercise rehabilitation adherence and associated factors in patients aged ≥75 years with hemorrhagic stroke: a mixed-methods study
Source: Front Neurol. 2026 Sep 15;17:1861509. doi: 10.3389/fneur.2026.1861509 (PMC13619532; doi:10.3389/fneur.2026.1861509)
Supplement: Supplementary file 1 [file Supplementary_file_1.docx]

| **Number** | **Core interview question** |
| --- | --- |
| 1 | Could you describe your usual exercise or rehabilitation activities? |
| 2 | Do you enjoy exercising? Do you think exercise is helpful to you? |
| 3 | Do you find it difficult to maintain regular exercise? What factors make it difficult for you to engage in exercise or rehabilitation? Why? |
| 4 | Who do you think influences your adherence to exercise or rehabilitation recommendations? How do they influence you? |
| 5 | Apart from these factors, are there any other reasons that make it difficult for you to follow the exercise recommendations provided by healthcare professionals? |
| 6 | Could you describe your feelings and experiences during exercise or rehabilitation? |

Note: The interview questions were used flexibly as a semi-structured guide. Follow-up questions and probes were used when appropriate to clarify participants' responses and explore relevant experiences in greater depth.
